# Meal-Sensing Signaling Pathways in Functional Dyspepsia

**DOI:** 10.3389/fnsys.2018.00010

**Published:** 2018-04-05

**Authors:** Amanda J. Page, Hui Li

**Affiliations:** ^1^Adelaide Medical School, University of Adelaide, Adelaide, SA, Australia; ^2^South Australian Health and Medical Research Institute (SAHMRI), Adelaide, SA, Australia

**Keywords:** gastrointestinal tract, mechanosensation, chemosensation, pain, vagal afferents, functional dyspepsia

## Abstract

The upper gastrointestinal tract plays an important role in sensing the arrival, amount and chemical composition of a meal. Ingestion of a meal triggers a number of sensory signals in the gastrointestinal tract. These include the response to mechanical stimulation (e.g., gastric distension), from the presence of food in the gut, and the interaction of various dietary nutrients with specific “taste” receptors on specialized enteroendocrine cells in the small intestine culminating in the release of gut hormones. These signals are then transmitted to the brain where they contribute to food intake regulation by modulating appetite as well as feedback control of gastrointestinal functions (e.g., gut motility). There is evidence that the sensitivity to these food related stimuli is abnormally enhanced in functional dyspepsia leading to symptoms such nausea and bloating. In addition, these gut-brain signals can modulate the signaling pathways involved in visceral pain. This review will discuss the role of gut-brain signals in appetite regulation and the role dysregulation of this system play in functional dyspepsia.

## Introduction

The gastrointestional (GI) tract plays an important role in sensing the arrival, amount and chemical composition of a meal. However, exaggerated perception of a meal can have significant implications, across the lifespan, for eating-related disorders such as functional dyspepsia (Feinle-Bisset, 2016). The GI tract is richly innervated by sensory nerves that convey information to the central nervous system (CNS) where it is processed and gut reflexes are coordinated with behavioral responses and sensations such as satiety, fullness, nausea, bloating and pain. Sensory nerves that originate in the nodose and jugular ganglia project to the gut via the vagal nerves, whereas, thoracolumbar spinal afferent neurons project to the GI tract via the splanchnic nerves. While most studies have focussed on the role of spinal afferents in mediating visceral pain (Gebhart and Ness, 1991; Mayer and Gebhart, 1994; Vermeulen et al., 2014; Spencer et al., 2016) there is evidence to suggest that vagal afferent fibers play an important role in the modulation of nociception (Grundy, 1988; Randich and Gebhart, 1992; Holtmann et al., 1998; Wang et al., 2015). This review will focus on the role vagal afferent fibers may play in the symptoms associated with functional dyspepsia including pain.

## Vagal Afferent Innervation of the Gut

### Anatomy

The sensory nerves within the vagus originate from two different embryonic tissues. A proportion, of vagal sensory nerves (afferents), is derived from the embryonic placodes and the cell bodies are located in the nodose ganglia. The other population of vagal afferent cell bodies are located in the jugular ganglia. These afferents are derived from the embryonic neural crest similar to spinal sensory neurones. It has been hypothesized, based on the common neural crest origin of nociceptive spinal and vagal jugular sensory neurones, that the jugular ganglia supply the GI tract with nociceptive sensory nerves (Yu et al., 2005).

In the GI tract, at least three distinct vagal afferent terminals are present at specific locations within the gut wall, including the external muscular layers, the myenteric plexus and the mucosal lamina propria. Vagal afferent endings in the longitudinal and circular muscular layers consist of long trails of branching and interconnecting fibers embedded within the muscular layers and arranged in parallel to the muscle fibers (Berthoud and Powley, 1992; Phillips et al., 1997). As a consequence these nerve endings are known as intramuscular arrays (IMAs; Berthoud and Neuhuber, 2000). IMAs are located throughout the GI tract, however, the highest density is observed in the stomach wall and the sphincters throughout the gut (Wang and Powley, 2000). Given their morphology it has been hypothesized that IMAs could function as tension receptors monitoring the length of the muscle cells, however, there is no electrophysiological data to confirm IMAs are tension sensitive.

The most abundant type of vagal afferent terminal is the intraganglionic laminar endings (IGLEs) positioned in the myenteric plexus between the longitudinal and circular muscular layers (Berthoud and Powley, 1992; Fox et al., 2000). It is hypothesized that IGLEs detect distortion of the surrounding tissue (Zagorodnyuk et al., 2001). Their stimulus-response functions saturate within the physiological range (Sengupta et al., 1989), unlike spinal afferents which generally signal well above the physiological range (Sengupta et al., 1990).

A third population of vagal afferent has endings in the mucosal lamina propria where they are ideally situated to detect fine tactile mechanical stimuli (Page et al., 2002) or material absorbed across the mucosal epithelium or released from specialized cells (e.g., glucagon-like peptide-1 (GLP-1) from L-cells; Berthoud et al., 1995; Berthoud and Patterson, 1996; Williams et al., 1997). Specialization of these mucosal vagal afferent endings is region specific. In the stomach, the mucosal afferents detect the presence of luminal content via their response to mechanical and chemical stimulation. In the small intestine, the vagal afferent terminals are in close contact with the basal lamina and therefore ideally positions to detect absorbed nutrients and/or hormones and peptides released from epithelial cells or other nerve fibers.

Centrally, these vagal afferent nerve fibers project to the nucleus tractus solitarius (NTS) where the information they transmit is integrated with brainstem, limbic and hypothalamic signals to ultimately provide coordinated GI reflexes (e.g., motility and gastric emptying; Berthoud et al., 2004; Brookes et al., 2013) along with behavioral responses and sensations, such as fullness, satiety and bloating.

### Functional Properties of Vagal Afferents

Vagal afferents have been shown to respond to a number of different stimuli including nutrient and nutrient-related compounds, mechanical stimulation, temperature and osmolarity (Berthoud and Neuhuber, 2000). In this review we will focus on the mechanical and chemical properties of GI vagal afferent endings in the upper GI tract due to their possible role in functional dyspepsia.

As food is ingested the vagal afferents innervating the stomach respond to mechanical stimulation as the undigested food enters, fills and distends the stomach wall. There are two fundamental classes of mechanosensitive vagal afferent ending in the stomach according to location and response to mechanical stimuli (Page and Blackshaw, 1998; Page et al., 2002). Mucosal receptors are generally silent at rest and are sensitive to light stroking of the mucosa, generating a burst of action potentials each time the stimulus passes over the receptive field (Page and Blackshaw, 1998; Page et al., 2002). They are insensitive to distension and contraction of the gastric wall. There is evidence that they are important in the initiation of satiety, nausea and vomiting by chemical and osmotic stimuli (Andrews and Sanger, 2002). Although there is no direct evidence, mucosal receptors are thought to discriminate particle size and give negative feedback on the control of gastric emptying (Becker and Kelly, 1983; Tuleu et al., 1999); as a consequence food is not released from the stomach until sufficiently churned. Tension receptors often have a resting discharge that may be modulated in phase with ongoing contractions. They show slowly adapting responses to normal contractions and distension with a linear relationship to wall tension (Page and Blackshaw, 1998; Page et al., 2002). Tension receptors signal the level of gastric distension to the CNS, which is important not only in triggering reflexes controlling gastrointestinal function, but is also critical in signaling food intake and generating sensations such as satiety and fullness. Existing evidence indicates that both tension and mucosal receptors play distinct but complementary roles in the generation of mechanosensory satiety signals. The mechanosensitivity of both mucosal and tension sensitive afferents can be modulated by a variety of different mediators including gut hormones, such as ghrelin (Page et al., 2007; Kentish et al., 2012) found in specialized cells within the stomach wall, and adipokines, such as leptin (Kentish et al., 2013).

As gastric emptying occurs, nutrients enter the small intestine and interact with nutrient receptors on the surface of specialized cells within the intestinal mucosal layer. This initiates an intracellular cascade that culminates in the release of one or more gut hormones that can then activate vagal afferent endings that project along the length of the villi, ramifying beneath the epithelial layer (Powley and Phillips, 2011). There are numerous subclasses of enteroendocrine cells releasing a subset of mediators. These hormones, as described above, can act in a paracrine fashion on vagal afferent endings, act as true hormones coordinating activities within the gut (e.g., secretory function) or by acting in the brain via the circulation or can work by more than one of these pathways.

A subset of enteroendocrine hormones/mediators act as satiety factors largely through local effects on vagal afferent endings. These hormones include cholecystokinin (CCK), GLP-1 and peptide YY (PYY). CCK is released from I-cells located largely in the proximal small intestine. The release of CCK is mediated by the presence of luminal nutrients and both long-chain fatty acids and casein cause activation of mucosal afferents via a CCK-1 (a.k.a. CCK_A_) receptor dependent mechanism (Eastwood et al., 1998; Lal et al., 2001). Administration of CCK reduces food intake (Gibbs et al., 1973) and antagonists of the CCK-1 receptor increase food intake (Hewson et al., 1988) indicating that endogenous CCK has a role in the control of food intake. The satiety effects of CCK require an intact vagus (Blackshaw and Grundy, 1990).

GLP-1 is an incretin hormone released from intestinal L-cells (Lee et al., 1990). These cells have the ability to respond to a broad range of nutrients including digestion products of proteins, fats and carbohydrates (Elliott et al., 1993). The release of GLP-1 causes a decrease in food intake (Gutzwiller et al., 1999), stimulation of insulin release (Fridolf and Ahrén, 1991), reduction in glucagon secretion (Drucker, 2006) and a reduction in gastric emptying (Delgado-Aros et al., 2002). GLP-1 receptor is expressed in the nodose ganglia (Nakagawa et al., 2004) and has been localized on vagal afferent endings (Bucinskaite et al., 2009). Further, GLP-1 has been shown to increase gastric and jejunal vagal afferent activity (Bucinskaite et al., 2009; Gaisano et al., 2010) and activation of vagal afferents is thought to be the mechanism responsible for its effect on food intake and insulin release (Hayes et al., 2011). However, there is some debate on whether vagal afferents are the main pathway by which GLP-1 signals to the brain. Following subdiaphragmatic vagotomy GLP-1 induced changes in food intake were not affected if the GLP-1 was administered via the hepatic portal vein but abolished if administered intraperitoneally (Rüttimann et al., 2009). Therefore, local and circulating GLP-1 may have different effector locations. For example, the effects of GLP-1 on satiety and gastric emptying could also involve endocrine actions at central sites within the brainstem and hypothalamic nuclei (Turton et al., 1996; Imeryüz et al., 1997; Nagell et al., 2006; Nakade et al., 2006).

PYY, similar to GLP-1, is released from intestinal L-cells. However, the distribution of PYY positive cells is different from GLP-1 and although PYY is present throughout the intestine there are very low levels in the proximal small intestine with levels increasing substantially in the ileum and even more in the colon (Adrian et al., 1985). The release of PYY is regulated by direct contact with luminal nutrients or indirectly through CCK release in response to proximal exposure to fat (Greeley et al., 1989). Intraperitoneal administration of PYY_3–36_ has been shown to have an anorexigenic effect in rodents (Batterham et al., 2002), an effect completely abolished by subdiaphragmatic vagotomy (Abbott et al., 2005; Koda et al., 2005). This is further supported by the fact that PYY receptors (Y2) are expressed in intestinal vagal afferents (Burdyga et al., 2008). A blockade of the Y2 receptor has been shown to abolish the anorectic effects of PYY_3–36_ (Scott et al., 2005) and Y2 knockout mice exhibit hyperphagia (Naveilhan et al., 1999). Similar to GLP-1, the anorectic effects of PYY could be due to paracrine effects on vagal afferents, as described above, or via direct central activation by circulating PYY, or by both pathways (Zhang et al., 1997; Fetissov et al., 2004; Koda et al., 2005).

Therefore whilst capable of spontaneous activity the signaling of vagal afferents within the GI tract are modulated or activated by an array of substances released from specialized cells within the GI mucosa which allows for rapid communication in response to GI motility and nutrient content. If something is disrupted in this multilevel system it could have a serious impact on symptoms associated with functional dyspepsia.

## Functional Dyspepsia and Vagal Afferent Signaling

Functional dyspepsia is associated with enhanced GI sensitivity with no clear evidence of an organic cause (Tack et al., 2006). Functional dyspepsia affects about 20% of the population and significantly impairs their quality of life. Functional dyspepsia is meal related with about 80% of patients reporting that the symptoms are aggravated by ingestion of a meal (Bisschops et al., 2008). Hypersensitivity to mechanical stimulation of the stomach is frequent in functional dyspeptic patients, however, the underlying mechanisms for this hypersensitivity are unclear. Functional dyspepsia is also associated with delayed gastric emptying and reduced gastric accommodation after a meal (Tack et al., 1998; Sarnelli et al., 2003). Failure of the stomach to accommodate food is also linked to the increase in transient lower esophageal sphincter relaxations that occur in patients with gastroesophageal reflux disease (GERD; Pauwels et al., 2014). This may in part explain the overlap in functional dyspepsia and GERD patients (Pauwels et al., 2014) and be explained by enhanced gastric vagal afferent mechanosensitivity, although this still remains to be determined. There is some indication transient receptor potential (TRP) channels may be involved in the visceral hypersensitivity associated with functional gastrointestinal disorders (Balemans et al., 2017). The best characterized of these channels is TRP vanilloid receptor 1 (TRPV1) a voltage-gated outwardly rectifying cation channel activated by acidosis (pH < 6), noxious heat (Tominaga et al., 1998), exogenous irritants such as capsaicin (active component of hot chilli peppers; Caterina et al., 1997) and endocannabinoids such as anandamide (Zygmunt et al., 1999). TRPV1 is expressed in the nodose ganglia (Kentish et al., 2015) and activation of TRPV1 increases gastric vagal afferent excitability thereby signaling satiety whereas inhibition of TRPV1 does the converse (Bielefeldt and Davis, 2008). Capsaicin reduces food intake in humans (Yoshioka et al., 2001) and consumption of spicy capsaicin containing food was positively associated with scores of stomach fullness in functional dyspepsia patients (Lee et al., 2016). Further, there is hypersensitivity to capsaicin in patients with functional dyspepsia compared to healthy controls (Hammer et al., 2008). Therefore, it is possible that enhanced TRPV1 signaling in gastrointestinal vagal afferents plays a role in the symptoms associated with functional dyspepsia, however, further investigation is required. Unfortunately, unlike irritable bowel syndrome (IBS) a functional disorder of the lower GI tract, the majority of research on functional dyspepsia has occurred at the clinical level with very few basic research studies investigating the mechanisms driving hyper-perception of food related stimuli in the upper GI tract. This is mainly due to the lack of animal models for functional dyspepsia. An animal model would allow the investigation of the molecular mechanisms driving gastric vagal afferent hypersensitivity and possibly identify new targets for the treatment of functional dyspepsia.

Functional dyspepsia is further subdivided into two clinically distinct syndromes:

### Postprandial Distress Syndrome

**Figure 1:**
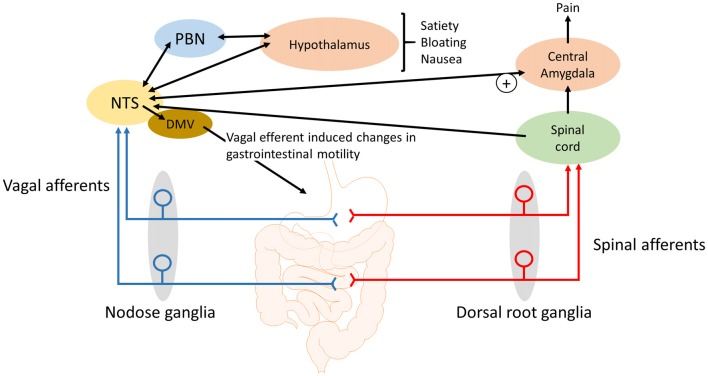
Schematic of how gastrointestinal vagal afferents can be involved in the symptoms associated with functional dyspepsia. For simplicity not all neural pathways and regions are illustrated. The NTS receives input from vagal afferents innervating the gastrointestinal tract. Distinct neural outputs from the NTS coordinate sensations such as satiety, bloating and nausea. Further, neural output from the NTS to the central amygdala can modulate the processing of nociceptive information, from the spinal cord and brainstem, within the central amygdala. NTS, nucleus tractus solitarius; DMV, dorsal motor nucleus of the vagus; PBN, Parabrachial nucleus.

In postprandial distress syndrome, feelings of fullness (satiety) occur early in the meal, preventing the completion of a normal size meal, and/or there is persistent feelings of bloating or nausea, with symptoms occurring after eating at least several times a week. Hypersensitivity to gastric distension occurs in 30%–40% of functional dyspeptic patients (Tack et al., 2001; Boeckxstaens et al., 2002). This alone could explain the early satiety and inability to complete a meal (Figure 1). Additional studies suggest that 60%–70% of patients with functional dyspepsia are hypersensitive to nutrients (Barbera et al., 1995a; Feinle et al., 2001). For example, duodenal infusion of a long chain lipid emulsion increases symptoms of fullness, nausea and bloating and increases responses to gastric distension in functional dyspepsia patients compared to healthy individuals (Barbera et al., 1995b). There is evidence that gut hormones, at least in part, mediate these effects. It has been demonstrated that the plasma concentration of CCK is elevated in functional dyspepsia patients compared to healthy controls (Pilichiewicz et al., 2008). In addition, exogenous administration of CCK enhances symptoms in functional dyspeptic patients (Chua et al., 1994), whereas, the CCK antagonist, dexloxiglumide, has been shown to reduce symptoms during gastric distension and duodenal lipid infusion (Feinle et al., 2001). In summary, lipid hypersensitivity in functional dyspepsia patients is mediated, at least in part, via CCK acting on CCK-1 receptors (Feinle et al., 2001). The hypersensitivity appears to be fat-specific with duodenal glucose having no effect on symptoms (Barbera et al., 1995a).

Other hormones may also play a role in the symptoms associated with functional dyspepsia. For example, plasma levels of the gastric hormone acyl ghrelin have been reported to be reduced in postprandial distress syndrome (Choi et al., 2016). A reduced acyl ghrelin level has been correlated with impaired gastric emptying (Shindo et al., 2009) which can lead to postprandial fullness and vomiting (Stanghellini et al., 1996); diagnostic symptoms for functional dyspepsia. Growth hormone secretagogue receptor type 1a, the receptor for ghrelin, is expressed in the nodose ganglia (Burdyga et al., 2006) and ghrelin has been shown to increase and decrease responses to distension in the jejunum (Murray et al., 2006) and stomach (Page et al., 2007) respectively. This suggests specific vagal afferent populations respond differently to ghrelin. The role of ghrelin effects on these different vagal afferent populations in the symptoms associated with functional dyspepsia remain to be determined. The gut hormone nesfatin-1 may also be involved. A recent publication, using a rat stress model of functional dyspepsia, has demonstrated that nesfatin-1 protein levels were increased in the gastric fundus of stressed compared with control rats (Jing et al., 2017). Nesfatin has been shown to increase the sensitivity of gastric vagal afferent mucosal receptors (Kentish et al., 2017) and therefore heightened perception to meal related stimuli in functional dyspepsia could be due to the elevated gastric nesfatin-1 levels and subsequent effects on vagal afferents. Further, it is uncertain whether other gut hormones, such as GLP-1 and PYY also play a role in the symptoms associated with functional dyspepsia (Lanzini et al., 2006; Pilichiewicz et al., 2008).

### Epigastric Pain Syndrome

In epigastric pain syndrome there is intermittent pain or a burning epigastrium at least once a week. The pain experienced by dyspeptic patients may be due to pathological alterations in gut function and/or the events in the gastrointestinal tract may be exaggerated in the brain. This could be due to either: (1) an increase in the sensitivity of peripheral afferent nerves and therefore an increase in central input; or (2) the central integration is abnormally high in functional dyspepsia (Malagelada, 2001; Holzer, 2002). Further, summation of afferent input may also play a role in the pain experienced by dyspeptic patients because unperceived electrical stimulation of mechano-insensitive jejunal afferents has been shown to increase the perception of distension to uncomfortable levels (Accarino et al., 2002).

Although it was considered that vagal afferents are not involved in abdominal pain, with spinal afferents playing the predominant role in pain transmission, there is growing awareness of a role for vagal afferents in visceral pain (Michl et al., 2001; Lamb et al., 2003). For example, there is a population of esophageal vagal afferents, namely nodose and jugular C fibers, which have a high threshold of activation (~30 mmHg; Yu et al., 2005, 2008). Unlike the activity of vagal tension receptors that saturate at innocuous intra-esophageal pressures, this population linearly encode esophageal distention in the noxious range (Yu et al., 2005). Therefore, it is possible that similar gastric nociceptive vagal afferents, thus far unidentified, may contribute to epigastric pain syndrome along with the spinal afferents. However, it has been reported that distension-sensitive gastric vagal afferent fibers encode gastric distension but do not have thresholds in what may be considered the noxious range (Ozaki et al., 1999).

Gastric acid plays a role in the pain associated with GERD, gastritis and peptic ulcers (Kang et al., 1986, 1989) and there is some evidence that the painful symptoms of functional dyspepsia may also involve gastric acid as a noxious stimuli. Although gastric acid secretion is in the normal range (Collen and Loebenberg, 1989) in dyspeptic patients there are some indications that the stomach and duodenum, in these patients, might be hypersensitive to acid (Son et al., 1997; Samsom et al., 1999). In addition, acid can sensitize mechanosensitive afferents, presumably vagal afferents, in the stomach (Coffin et al., 2001). In rats, exposure of the gastric mucosa to a gastric acid challenge has been shown to lead to a rapid rise in c-Fos expression in the NTS but not the spinal cord (Schuligoi et al., 1998; Michl et al., 2001; Danzer et al., 2004). In addition, the medullary c-Fos response to a gastric challenge is blocked by bilateral vagotomy indicating that chemo-nociceptive gastric mucosal afferent input to the NTS and area postrema is predominantly carried by vagal afferents (Schuligoi et al., 1998; Michl et al., 2001). Consistent with these observations, it has been demonstrated in rats that the visceromotor electromyographic response to a gastric acid challenge is abolished by vagotomy and not splanchnectomy (Lamb et al., 2003). Data indicate that the central vagal afferent input, in response to a gastric acid challenge, is initially processed in the medullary brain stem before information is passed to the lateral parabrachial nucleus (PBN), thalamic and hypothalamic paraventricular nuclei, supraoptic nucleus, central amygdala and the mediolateral habenula (Michl et al., 2001). However, there was no observed activation of the insular cortex (Michl et al., 2001), which suggests that the vagal afferent responses, to a gastric acid challenge, do not give rise to the perception of pain but instead lead to activation of subcortical brain nuclei involved in emotional, autonomic, behavioral and neuroendocrine responses to the noxious stimuli (Michl et al., 2001). The participation of vagal afferents in nausea, emesis and in cytokine-evoked illness responses (Goehler et al., 2000; Konsman et al., 2002) corroborate this view of vagal afferent involvement in abdominal nociception (Traub et al., 1996). However, in addition to the emotional response to pain there is accumulating evidence for the role of the central amygdala in pain modulation. The latero-capsular region of the central amygdala has been defined as the “nociceptive amygdala” due to the high content of neurones that process nociceptive information from the spinal cord and brainstem (Neugebauer et al., 2004). As previously stated, there are direct projections from the NTS to the central amygdala (Ricardo and Koh, 1978; Zardetto-Smith and Gray, 1990) and systemically administered CCK has been shown to activate central amygdala neurons (Myers and Rinaman, 2002). CCK is unable to cross the blood-brain barrier (Passaro et al., 1982) and it has been demonstrated that systemic CCK enhances visceral pain responses to colorectal distension in rats in a vagal afferent dependent manner (Wang et al., 2015). It is known that plasma CCK levels are elevated in functional dyspepsia patients compared to healthy controls (Pilichiewicz et al., 2008) and that endogenously released CCK acts on vagal afferent mucosal terminals in the small intestine (Lal et al., 2001). Further, a CCK antagonist reduced symptoms to gastric distension in functional dyspeptic patients (Feinle et al., 2001) suggesting that in these patients CCK enhanced gastric vagal afferent tension receptor responses to distention stimuli. Therefore, it is conceivable that the observed hypersensitivity of gastric and small intestinal vagal afferents (Feinle et al., 2001) will augment visceral pain responses originating in the upper GI tract (Figure 1). However, this remains to be determined. Nevertheless, it seems likely that vagal afferents not only mediate the emotional, autonomic, behavioral and neuroendocrine responses but also the pain responses (Figure 1).

In summary, gastrointestinal vagal afferents play an important role in sensing the arrival, amount and chemical composition of a meal. However, exaggerated vagal afferent signaling can have significant implications for GI disorders, such as functional dyspepsia. Along with the vagal afferent function associated feelings in postprandial distress syndrome, such as early satiety, fullness and bloating, the hypersensitivity of vagal afferent responses to mechanical and chemical stimuli could also lead to central modulation of pain pathways and therefore play a role in the pain experienced in epigastric pain syndrome. However, further research is required to fully elucidate the role of gastrointestinal vagal afferents in these syndromes.

## Author Contributions

Both AJP and HL contributed equally to the mini review.

## Conflict of Interest Statement

The authors declare that the research was conducted in the absence of any commercial or financial relationships that could be construed as a potential conflict of interest.
